# Pulmonary Arterial Remodeling Is Related to the Risk Stratification and Right Ventricular-Pulmonary Arterial Coupling in Patients With Pulmonary Arterial Hypertension

**DOI:** 10.3389/fphys.2021.631326

**Published:** 2021-05-03

**Authors:** Juan C. Grignola, Enric Domingo, Manuel López-Meseguer, Pedro Trujillo, Carlos Bravo, Santiago Pérez-Hoyos, Antonio Roman

**Affiliations:** ^1^Pathophysiology Department, Hospital de Clínicas, Facultad de Medicina, Universidad de la República, Montevideo, Uruguay; ^2^Area del Cor, Hospital Vall d’Hebron, Barcelona, Spain; ^3^Physiology Department, School of Medicine, Universitat Autonoma, Barcelona, Spain; ^4^Department of Pneumology, Hospital Vall d’Hebron, Barcelona, Spain; ^5^Ciberes, IS Carlos III, Madrid, Spain; ^6^Centro Cardiovascular Universitario, Hospital de Clínicas, Facultad de Medicina, Universidad de la República, Montevideo, Uruguay; ^7^Unidad de Estadística, Vall d’Hebron Institut de Recerca, Barcelona, Spain

**Keywords:** pulmonary arterial stiffness, right ventricular-arterial coupling, pulmonary arterial hypertension, intravascular ultrasound, risk stratification

## Abstract

**Background:**

Pulmonary arterial (PA) stiffness has an essential contribution to the right ventricular (RV) failure pathogenesis. A comprehensive and multiparameter risk assessment allows predicting mortality and guiding treatment decisions in PA hypertension (PAH). We characterize PA remodeling with intravascular ultrasound (IVUS) in prevalent and stable patients with PAH according to the ESC/ERS risk table and analyze the RV-PA coupling consequences.

**Methods:**

Ten control subjects and 20 prevalent PAH adult patients underwent right heart catheterization (RHC) with simultaneous IVUS study. We estimated cardiac index (CI), pulmonary vascular resistance, and compliance (PVR, PAC) by standard formulas. From IVUS and RHC data, PA diameter, wall thickness/luminal diameter ratio, and indexes of stiffness (pulsatility, compliance, distensibility, incremental elastic modulus - Einc-, and the stiffness index β) were measured. We evaluated RV-PA coupling by the ratio of tricuspid annular plane systolic excursion to systolic pulmonary arterial pressure (TAPSE/sPAP). The individual average risk was calculated by assigning a score of 1 (low-risk -LR-), 2 (intermediate-risk -IR-), and 3 (high-risk -HR-) for each of seven variables (functional class, six-minute walking test, brain natriuretic peptide, right atrial area and pressure, CI, and PA oxygen saturation) and rounding the average value to the nearest integer.

**Results:**

All PA segments interrogated showed increased vessel diameter, wall cross-sectional area (WCSA), and stiffness in patients with PAH compared to control subjects. 45% corresponded to LR, and 55% corresponded to IR PAH patients. The different measurements of PA stiffness showed significant correlations with TAPSE/sPAP (*r* = 0.6 to 0.76) in PAH patients. The IR group had higher PA stiffness and lower relative WCSA than LR patients (*P* < 0.05), and it is associated with a lower PAC and TAPSE/sPAP (*P* < 0.05).

**Conclusion:**

In prevalent PAH patients, the severity of proximal PA remodeling is related to the risk stratification and associated with PAC and RV-PA coupling impairment beyond the indirect effect of the mean PA pressure. The concomitant assessment of IVUS and hemodynamic parameters at diagnosis and follow-up of PAH patients could be a feasible and safe tool for risk stratification and treatment response of the PA vasculopathy during serial hemodynamic measurements.

## Introduction

Emerging evidence supports the idea that vascular stiffening in the pulmonary arterial bed can precede the development of pulmonary hypertension (PH) as an early disease marker and promotes pulmonary vascular remodeling that ultimately leads to right ventricular (RV) failure (Sanz et al., 2009; Wang and Chesler, 2011; Lammers et al., 2012). Measurements of pulmonary vascular stiffness are considered to be superior to other hemodynamic parameters in predicting mortality (Mahapatra et al., 2006). However, the exact role of vascular stiffening in the development and progression of PH is lacking due to insufficient understanding of the spatiotemporal development of pulmonary vascular stiffness (Dabral and Pullamsetti, 2017). It has been proposed that the crosstalk between the proximal-elastic and distal-muscular pulmonary arteries (PAs) play a role in the PH progression. Stiffer proximal large PA will enhance transmission of flow and pressure pulsatility (shear and barotrauma) to the distal low impedance (high compliance/low resistance) PAs, causing its muscularization and remodeling (Dieffenbach et al., 2018). Muscularization of distal PAs promotes increased mean arterial pressures, resulting in extensive vessel wall remodeling, stiffening the large PAs in a positive feedback cycle of pathologic vascular remodeling. Finally, vascular stiffening may promote local remodeling through alterations in gene expression and cellular behaviors in response to the local mechanical microenvironment (“mechanobiological feedback”) (Bertero et al., 2015). Arterial stiffness can be obtained by assessing the relation between changes in arterial pressure (“stress”) and changes in arterial volume, cross-sectional area, or diameter (“strain”). Both cardiac magnetic resonance and intravascular ultrasound (IVUS) are increasingly used to evaluate PA dimensions and different arterial stiffness parameters (Rodes-Cabau et al., 2003; Sanz et al., 2009; Lau et al., 2012; Shen et al., 2016).

The prognosis of PAH patients is determined mainly by RV adaptation to increased afterload. Accurate risk assessment is essential in PAH to optimize treatment decisions aiming to slow disease progression and improve outcomes (Galie et al., 2018). Nowadays, achieving a low-risk status has been proposed as a potential surrogate outcome in PAH clinical trials (Weatherald et al., 2018). There are different approaches to assessing risk in PAH by using different baseline and follow-up parameters, including clinical, functional, exercise, noninvasive and invasive variables combined in formulas or calculators (Benza et al., 2012, 2018; Hoeper et al., 2018). The 2015 European Society of Cardiology (ESC)/European Respiratory Society (ERS) PH guidelines propose a comprehensive and multidimensional risk strategy using several modifiable risk factors based on expert opinion and validated by three independent contemporary European PAH cohorts recently (Boucly et al., 2017; Hoeper et al., 2017; Kylhammar et al., 2018).

PA stiffness is an important factor governing the RV afterload and the RV-PA coupling. The increased PA stiffness leading to higher RV pulsatile workload reduced contractile performance and impaired RV-PA coupling. Guazzi et al. (2013) first proposed tricuspid annular plane systolic excursion normalized by pulmonary artery systolic pressure (TAPSE/sPAP) as a noninvasive measurement RV-PA coupling and an independent predictor of survival in patients with heart failure with and without PH. In the last few years, Tello et al. (2019b) proposed TAPSE/sPAP ratio as a surrogate of the gold standard measure of RV-PA coupling (invasive pressure-volume loop-derived end-systolic/arterial elastance [Ees/Ea] ratio) in PAH patients. They also showed that TAPSE/sPAP was associated with hemodynamics, functional class, and outcome in patients with PAH, proposing the possible added value of TAPSE/sPAP in current risk assessment strategies for PAH (Tello et al., 2018).

The present study aimed to characterize PA remodeling with IVUS in prevalent patients with PAH according to the disease severity assessed by a multiparametric risk stratification approach and analyze the RV-PA coupling consequences. We hypothesized that the pulmonary arterial remodeling (structural and functional wall properties) is related to the estimated risk score (ESC/ERS risk table) and is associated with the RV-PA impairment coupling in prevalent PAH patients.

## Materials and Methods

### Study Population

The study was conducted according to the principles expressed in the Declaration of Helsinki. The local institutional ethics committee approved the study, and all patients provided written informed consent.

Twenty prevalent PAH patients clinically stable in NYHA functional class I-III were recruited between April 2016 and October 2017. Selection patients were consecutive patients attending the Pulmonary Hypertension Unit outpatient of Hospital Universitari Vall d’Hebron that were stable and accepted the protocol. A stable patient was defined by no clinical status changes and PAH-specific medication in the previous 6 months. The only exclusion criteria were refusal to participate in the study or having PAH in functional class IV. PH was defined as mPAP greater or equal than 25 mmHg.

The same physician performed clinical assessment of patients who categorized functional class and certified clinical stability.

We compared the RV afterload (PVR and PAC) and PA remodeling indices of PAH patients with a historical control group of subjects (*n* = 10) referred for cardiac catheterization due to clinically suspected PH and in whom PH and other respiratory and cardiovascular diseases were discarded (Domingo et al., 2017).

### Hemodynamic and IVUS Examination

All PAH patients underwent right heart catheterization (RHC) in the supine position after 12 h of fasting and breathing room air. A 7F Swan-Ganz catheter (Edwards Lifesciences, United States) was inserted into a brachial or femoral vein, and a 5F end-hole catheter was inserted into a radial artery. Catheters were connected to fluid-filled transducers and zeroed at the atmospheric pressure. Hemodynamic measurements included right atrial pressure, systolic (sPAP), diastolic (dPAP), mean (mPAP), and pulse PA pressure (pPAP), PA occlusion pressure (PAOP), cardiac output and cardiac index (Fick method), pulmonary vascular resistance (PVR, calculated as mPAP minus PAOP divided by the cardiac output), PA oxygen saturation, and pulmonary arterial compliance (PAC, estimated as stroke volume divided by pPAP).

**FIGURE 1:**
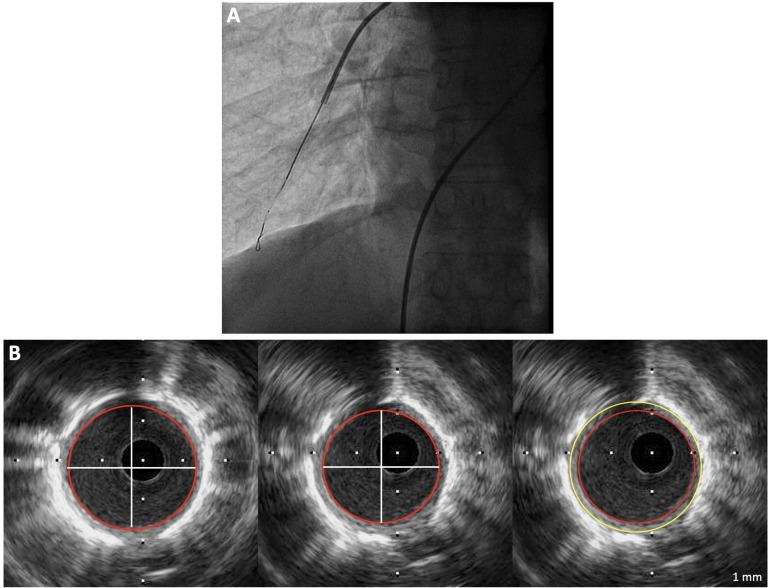
**(A)** Intravascular ultrasound probe positioning with X-Ray control. **(B)** Measurements of maximum systolic area, minimum diastolic area, and wall cross-sectional area in a pulmonary arterial hypertension patient (Diameter: 2.8 mm; pulsatility 31%; WCSA: 4.6 mm^2^; relative WCSA: 18%).

After the hemodynamic study was completed, a guiding catheter (Boston Scientific MPA Convey 7F) was advanced into different pulmonary arterial tree locations for IVUS image acquisition, as seen in Figure 1. IVUS examination of the PAs was performed using a 40 MHz catheter (Opticross, Boston Scientific, United States) advanced over a 0,014” guidewire with a 10–15 mm scan area and axial resolution of 43 mm. Selective pulmonary angiography was performed to determine the anatomical position of the most distal, least-branching segment of the PA with good image quality (defined as the complete circumferential demarcation of the intima and wall to adventitia boundaries) (Figure 1). We attempted to image at least four different pulmonary locations across the lungs, including upper and lower lobes of indistinct lungs. The images were obtained and stored in digital format. Both diastolic and systolic cross-sectional areas and the diameter of the studied segment were analyzed post-procedure by two independent observers unaware of clinical and hemodynamic findings.

Intra and interobserver validation of IVUS-derived measurements of total PA area, luminal area, and diameter have previously been reported (Rodes-Cabau et al., 2003; Grignola et al., 2013). All pressure measurements and IVUS images were acquired in end-expiration.

### Data Analysis

Total vessel area was defined by the area circumscribed by the bright elastic lamina; wall cross-sectional area (WCSA) was defined as the total vessel area minus luminal cross-sectional area (LCSA) during diastole; and %WCSA as WCSA divided by total vessel area × 100 (Gussenhoven et al., 1989). The mean vessel diameter of the segments was obtained from the LCSA.

We estimated the wall thickness/lumen diameter ratio (h/D) from the equations of Bramwell-Hill and Moens-Korteweg that provide the link between the local PWV and the distensibility (corresponding to the inverse of the pressure-strain modulus, E_P_), the wall thickness (h), and lumen diameter (D) of the artery (Bramwell and Hill, 1922).

$$\left(\mathrm{Bramwell}-\mathrm{Hill}\right)\mathrm{PWV}=\sqrt{\frac{{\text{E}}_{\text{P}}}{\rho }}$$

$$\left(\mathrm{Moens}-\mathrm{Korteweg}\right)\mathrm{PWV}=\sqrt{\frac{\text{h}\times \text{Einc}}{\text{D}\times \rho }}$$

$$\frac{\text{h}}{\text{D}}=\frac{{\text{E}}_{\text{P}}}{\text{Einc}}$$

where Einc corresponds to the incremental elastic modulus, and ρ corresponds to blood density (Table 1).

**TABLE 1:** Indices of local pulmonary arterial stiffness.

Parameter	Formula	Notes
Pulsatility, %	$\frac{\left(\mathrm{sLCSA}-\mathrm{dLCSA}\right)}{\text{dLCSA}}\times 100$	Relative change in lumen area during cardiac cycle
Compliance Coefficient, mm^2^/mmHg	$\frac{\left(\mathrm{sLCSA}-\mathrm{dLCSA}\right)}{\text{pPAP}}$	Absolute change in lumen area for a given change in pressure
Distensibility Coefficient, %/mmHg	$\frac{\left(\mathrm{sLCSA}-\mathrm{dLCSA}\right)}{\text{dLCSA}\times \text{pPAP}}\times 100$	Relative change in lumen area for a given change in pressure
Incremental elastic modulus (Einc), kPa	$3\times \left(1+\frac{\text{LCSA}}{\text{WCSA}}\right)\times {\text{E}}_{\text{P}}$	Elastic modulus defined over a particular stress or strain range
Local Pulse Wave Velocity (PWV), m/s	$\sqrt{\frac{{\text{E}}_{\text{P}}}{\rho }}$	Pulse generated by the ejecting heart propagates through the arterial wall with a given speed, the pulse wave velocity*
Stiffness index β	$\frac{\mathrm{Ln}\left(\frac{\text{sPAP}}{\text{dPAP}}\right)}{\left(\mathrm{sLCSA}-\mathrm{dLCSA}\right)/\mathrm{dLCSA}}$	Slope of the function between distending arterial pressure and arterial distension

dLCSA and sLCSA: diastolic and systolic lumen cross-sectional area, respectively; sPAP, dPAP, and pPAP: systolic, diastolic and pulse pulmonary arterial pressure, respectively; E_P_: pressure-strain modulus; ρ: blood density (1.055 g/cm^3^) (*, according to Bramwell-Hill equation for a uniform tube) (Bramwell and Hill, 1922).

Vessel diameter, h/D ratio, WCSA, and %WCSA were used to characterize structural PA remodeling.

Table 1 defines various indices used to assess local functional PA stiffness obtained from the invasive pressure and ultrasound measurements. We assumed that the cross-section of the artery is circular, with isotropic and incompressible wall (Laurent et al., 2006).

### Risk Stratification

Based on the ESC/ERS PH recommendation about the multidimensional risk stratification of PAH patients, we used a risk model of seven modifiable variables: functional class, six-minute walking test (6MWT), brain natriuretic peptide (BNP), right atrial area and pressure, cardiac index and PA oxygen saturation (Galie et al., 2016). The individual global risk was calculated by assigning a score of 1, 2, and 3 for each variable (1 = low-risk, 2 = intermediate-risk, and 3 = high-risk). Dividing the sum of all grades by the number of variables rendered a mean grade. The mean grade was rounded to the nearest integer, which was used to define the patient’s risk (Hoeper et al., 2017; Kylhammar et al., 2018).

We obtained the right atrial area and the ratio of TAPSE/sPAP by a transthoracic echocardiogram performed following the recommendations of the European Association of Echocardiography (Galderisi et al., 2011).

6MWT, plasma sample to dose BNP, and transthoracic echocardiography were taken on the same day of the RHC; therefore, all the seven variables included in the risk score were obtained concomitantly.

In order to carry out a more in-depth analysis of risk stratification, we took into account the number of parameters at low-risk grade (French approach in low-risk patients) for the low-risk group and the advanced risk stratification strategy described by Yogeswaran et al. for the intermediate-risk group who distinguished low-intermediate and high-intermediate risk according to TAPSE/sPAP ratio (TAPSE/sPAP ≥ 0.24 mm/mmHg and TAPSE/sPAP < 0.24 mm/mmHg, respectively) (Boucly et al., 2017; Yogeswaran et al., 2020).

### Statistical Analysis

Data are expressed as mean ± SD. If continuous variables were found not to follow a normal distribution (Shapiro-Wilk test), they were expressed as median with the interquartile range (IQR) and were compared using unpaired Mann-Whitney U-test. Fischer’s exact test or Chi-squared test was used to compare categorical variables, expressed as the absolute number or percentage. Significant differences between control subjects and PAH risk groups were analyzed using one-way ANOVA. The association between structural (vessel diameter and WCSA) and functional vascular remodeling (Einc) and between PA stiffness indices and TAPSE/sPAP ratio were explored using linear regression analysis (Pearson coefficient). All tests were 2-tailed, and a *P* value of 0.05 was considered statistically significant. Statistical analyses were performed with the statistical package SPSS for Windows (version 21.0, SPSS Inc., Chicago, IL, United States).

## Results

### Demographic, Clinical, Hemodynamic, and Risk Profile

All patients belonged to group I and were idiopathic PAH. At the time of the inclusion, eight patients received monotherapy (40%), and twelve patients received combined therapy (60%) (eight patients with dual therapy and four with triple therapy) without significant differences between risk groups. The pulmonary vasodilator drugs used were distributed as follows: ambrisentan (8), bosentan (3), diltiazem (3), epoprostenol (4), inhaled iloprost (1), macitentan (2), sildenafil (8), tadalafil (6), selexipag (1).

Patients’ demographic, humoral, echocardiographic, and hemodynamic data are summarized in Table 2. The age and body mass index of PAH subjects (50 ± 10 years; 23.9 ± 4.9 kg/m^2^) and control subjects (51 ± 6 years; 23.6 ± 3.8 kg/m^2^) were well matched. Women predominate in the PAH group compared to control subjects (95 versus 60%, *P* < 0.05). 45% of patients corresponded to low risk and 55% to intermediate risk. None were at high risk. Compared with intermediate-risk, low-risk patients showed higher walking distance, stroke volume index, PAC and PA oxygen saturation, and a lower BNP concentration (*P* < 0.05).

**TABLE 2:** Summary of demographic, humoral, echocardiographic, and hemodynamic data.

	Total (n = 20)	Low-Risk (n = 9)	Intermediate-Risk (n = 11)	P-value
Age (years)	50 ± 10	45 ± 10	53 ± 10	0.091
Sex (n M / F)	1 / 19	0 / 9	1 / 10	1.000
BMI (kg/m^2^)	23.9 ± 4.9	21.9 ± 2.4	25.6 ± 5.8	0.085
BSA (m^2^)	1.66 ± 0.18	1.68 ± 0.19	1.64 ± 0.19	0.663
FC (n I-II / III)	18 / 2	9 / 0	9 / 2	0.479
6MWT (meters)	417 ± 100	471 ± 106	365 ± 76	0.049
BNP (ng/L)	47 (32–75)	35 (20–46)	60 (47–250)	0.016
RA area (cm^2^)	19 (16–22)	18 (16–20)	20 (18–24)	0.278
TAPSE (mm)	18.5 ± 3.0	18.3 ± 2.7	18.7 ± 3.3	1.000
TAPSE/sPAP (mm/mmHg)	0.25 ± 0.08	0.30 ± 0.08	0.21 ± 0.06	0.023
mPAP (mmHg)	48 ± 15	43 ± 10	53 ± 18	0.194
pPAP (mmHg)	44 ± 16	39 ± 11	49 ± 18	0.186
CI (L/min/m^2^)	2.0 ± 0.4	2.2 ± 0.3	1.9 ± 0.5	0.162
HR (bpm)	68 ± 10	67 ± 9	70 ± 11	0.569
SVI (mL/m^2^)	28 ± 5	33 ± 6	26 ± 4	0.033
RA pressure (mmHg)	8.9 ± 4.0	7.0 ± 3.2	11 ± 4.5	0.103
PAOP (mmHg)	11.2 ± 3.2	11.8 ± 2.9	10.4 ± 3.7	0.562
PVR (Wood unit)	11 ± 5.6	9.7 ± 4.7	13.0 ± 5.3	0.167
PAC (mL/mmHg)	1.1 (0.7–1.4)	1.3 (1.0–2.0)	0.8 (0.7–1.1)	0.045
RC time (sec)	0.66 ± 0.14	0.68 ± 0.16	0.64 ± 0.14	0.556
PA oxygen saturation (%)	68 (63–70)	70 (68–74)	64 (60–68)	0.001

*Data are presented as mean ± SD or median (interquartile range). BMI, body mass index; BNP, brain natriuretic peptide; BSA, body surface area; CI, cardiac index; FC, NYHA functional class; HR, heart rate; mPAP and pPAP, mean and pulse pulmonary arterial pressure, respectively; PAC, pulmonary arterial compliance; PAOP, pulmonary arterial occlusion pressure; PVR, pulmonary vascular resistance; RA, right atrial; SVI, stroke volume index; 6MWT, six-minute walking test; TAPSE/sPAP, tricuspid annular plane systolic excursion normalized by pulmonary artery systolic pressure.*

**FIGURE 2:**
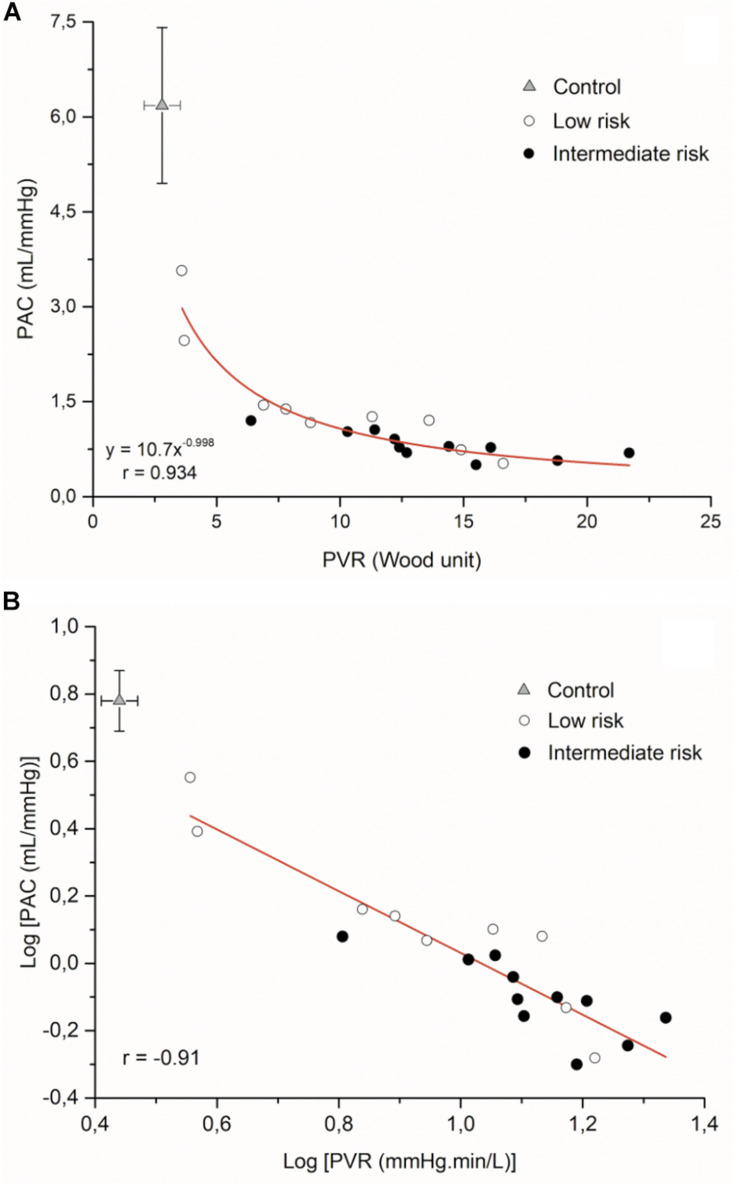
**(A)** Inverse relationship of PVR-PAC relationship and **(B)** Log PVR-log PAC plot for all patients (*n* = 9 low-risk patients; *n* = 11 intermediate-risk patients) (The PVR and PAC values of control patients are expressed by mean ± SD, *n* = 10). PAC, pulmonary arterial compliance; PVR, pulmonary vascular resistance).

Figure 2A displays the hyperbolic relationship of PVR and PAC of all patients, showing that low-risk subjects are at the upper and left end of the curve due to the higher PAC. PAH patients showed greater PVR (11 ± 5.6 versus 2.8 ± 0.74 Wu) and lower PAC (1.0 ± 0.4 versus 6.2 ± 1.1 mL/mmHg) compared to control group (*P* < 0.05). The logarithmically transformed PVR-PAC plot showed that linear regression slope was −0.91, indicating that PVR and PAC are tightly and inversely coupled (Figure 2B).

Based on the French low-risk approach, the number of variables in low-risk grade among low-risk patients showed the following distribution: nobody had all variables in low-risk grade, one patient had six variables, five patients had five parameters, and three patients showed only four parameters in low-risk grade.

Intermediate-risk patients had a reduced TAPSE/sPAP ratio than low-risk patients because a concomitant increase in contractility did not accompany the increase in afterload with the disease severity. Based on the advanced risk stratification proposed by Yogeswaran et al., five patients were in low-intermediate risk, and six patients were in high-intermediate risk.

### IVUS Measurements: PA Remodeling According to the Global Risk Assessment

As shown in Table 3, PA structural dimensions and stiffness indexes were higher in the patients with PAH than the control group (*P* < 0.05).

**TABLE 3:** Pulmonary arterial geometric (structural) and stiffness (functional) data.

	Total (*n* = 20)	Low-Risk (*n* = 9)	Intermediate- Risk (*n* = 11)	Control (*n* = 10)	*P*_1–2_	*P*_2–3_	*P*_1–3_
Diameter (mm)	4.4 (3.4–5.9)	3.7 (3.2–4.9)	4.9 (3.8–7.1)	3.8 (3.5–3.9)	0.002	0.009	0.385
h/D × 10^–2^	5.8 ± 0.9	5.9 ± 0.8	5.8 ± 1.1	2.4 ± 0.3	0.896	0.000	0.000
WCSA (mm^2^)	5.3 (4.7–6.5)	5.0 (4.4–5.5)	5.9 (5.1–7.1)	1.0 (0.8–1.2)	0.001	0.000	0.000
Relative WCSA (%)	15 ± 3	17 ± 2	13 ± 3	1.4 ± 1.3	0.000	0.000	0.000
Pulsatility (%)	30 ± 10	35 ± 11	25 ± 8	52 ± 8	0.031	0.000	0.001
CC (mm^2^/mmHg)	0.08 (0.06–0.1)	0.09 (0.06–0.12)	0.08 (0.06–0.1)	0.56 (0.37–0.69)	0.760	0.000	0.000
DC (%/mmHg)	0.52 (0.35–0.66)	0.61 (0.49–1.2)	0.35 (0.32–0.57)	4.54 (3.8–6.2)	0.020	0.000	0.000
PWV (m/s)	4.9 ± 1.2	4.2 ± 1.0	5.4 ± 1.1	1.6 ± 0.3	0.017	0.000	0.000
Einc (kPa)°	519 ± 292	344 ± 146	662 ± 325	116 ± 23	0.039	0.000	0.000
Stiffness index β	5.5 ± 2.9	3.6 ± 2.3	6.1 ± 2.8	1.4 ± 0.5	0.038	0.000	0.017

*Data are presented as the mean ± SD or median (interquartile range). CC, cross-sectional compliance coefficient; DC, cross-sectional distensibility coefficient; Einc, incremental elastic modulus; h/D, wall thickness/lumen diameter ratio; PWV, local pulse wave velocity. (°, 1 kPa = 7.5 mmHg) (P_1–2_, low-risk versus intermediate-risk; P _2–3_, intermediate-risk versus control; P _1–3_, low-risk versus control).*

A total of 85 PA segments were studied in PAH patients (4.2 seg/patient) with a median luminal diameter of 4.4 mm (IQR 3.4–5.9). Thirty-seven PA segments corresponded to low-risk patients [3.7 (3.2–4.9) mm] and forty-eight to intermediate-risk patients [4.9 (3.8–7.1) mm]. All PA segments interrogated in low-risk patients were from inferior lobes (59% left lung and 41% right lung). Among the forty-eight segments corresponding from intermediate-risk patients, thirty-six were interrogated from the inferior lobes, and twelve were interrogated from non-inferior lobes (62% left lung and 38% right lung). No significant differences were found in the diameter [5.5 (3.8–7.2) vs. 4.6 (3.7–6.5) mm], absolute WCSA [(6.1 (5.2–7.3) vs. 5.2 (4.9–6.9) mm^2^], and relative WCSA (13.1 ± 4.6 vs. 11.9 ± 5.6 %) of the vessels from the lower lobes versus the middle/upper lobes. However, the Einc of the PA segments from inferior lobes were lower than from the non-inferior lobes (567 ± 370 vs. 913 ± 580 kPa; *P* < 0.05).

**FIGURE 3:**
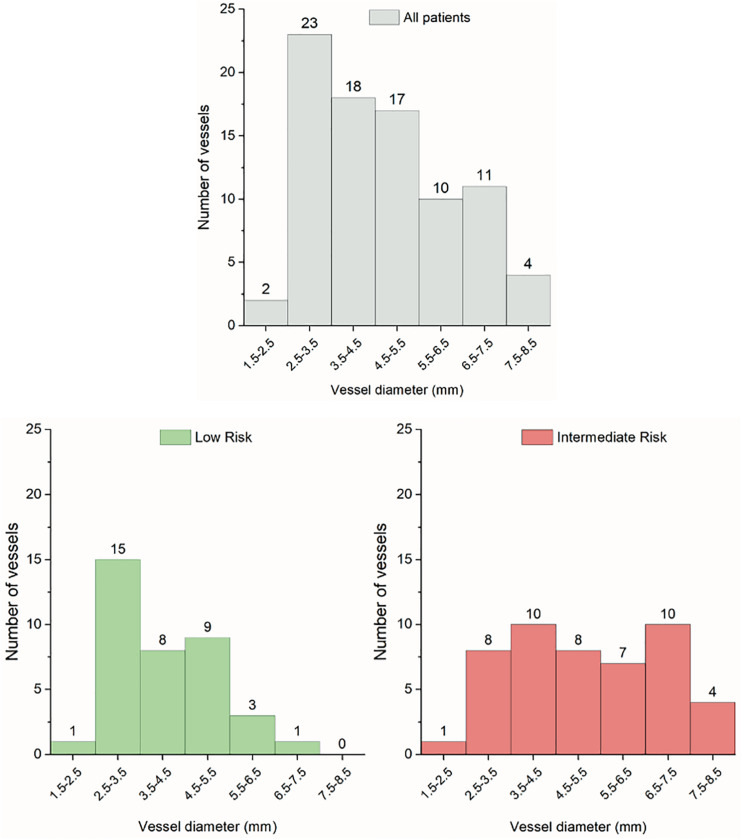
Histograms of the pulmonary artery segments from all patients (*n* = 85), low-risk (*n* = 37) and intermediate-risk patients (*n* = 48).

Figure 3 shows the distribution of the vessel diameters studied in the entire cohort and both risk groups. The segments of the low-risk patients are concentrated on smaller diameters than the segments of intermediate-risk patients distributed more homogeneously and extensively, reaching values greater than 5.5 mm in up to 44% compared to 11% in the low-risk group. Despite similar PAP, PVR, and h/D ratio, intermediate-risk subjects had lower PAC, higher diameter, absolute WCSA, and PA stiffness (except compliance coefficient) than patients with low-risk subjects (Table 3).

**FIGURE 4:**
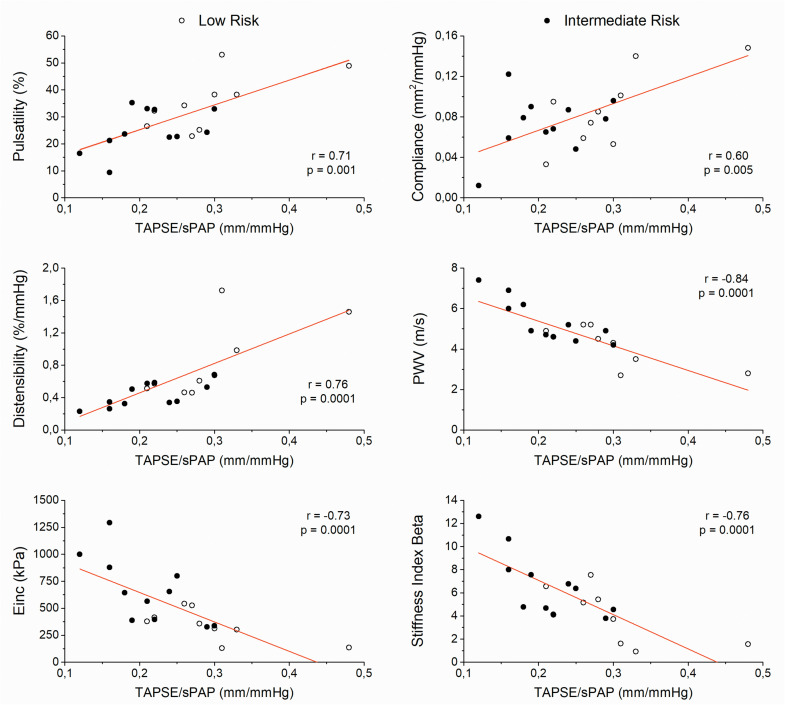
Scatter plots between pulmonary arterial stiffness indices and the ratio of tricuspid annular plane systolic excursion to systolic pulmonary artery pressure (TAPSE/sPAP) (Einc, incremental elastic modulus; PWV, local pulse wave velocity). (*n* = 9 low-risk patients; *n* = 11 intermediate-risk patients).

All PA stiffness indices were significantly correlated with TAPSE/sPAP ratio (Figure 4).

**FIGURE 5:**
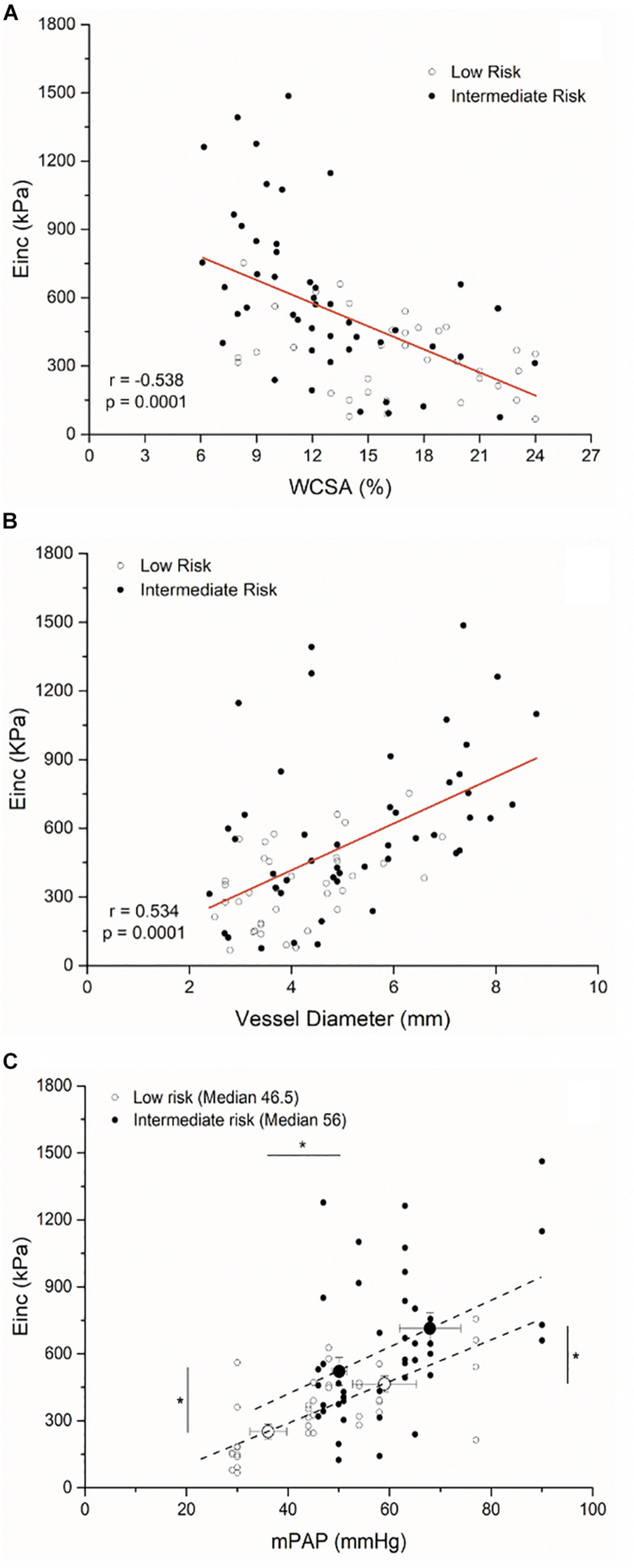
Scatter plot between the incremental elastic modulus (Einc) and the relative wall cross-sectional wall (WCSA) **(A)**, the vessel diameter **(B)**, and the mean pulmonary arterial pressure (mPAP) **(C)** of all PA segments interrogated (*n* = 37 low-risk patient segments; *n* = 48 intermediate-risk patient segments). (*P < 0.05 differences between low-risk and intermediate-risk patients of the Einc and mPAP below and above the median values, respectively).

From the pooled data of all segments, there was a significant correlation between Einc and relative WCSA (*r* = −0.538; *P* < 0.05) and between Einc and vessel diameter (*r* =−0.534; *P* < 0.05) (Figures 5A,B). We estimated the median values of the Einc PA segments and mPAP of each risk group. Einc values below and above the median values showed an upward displacement from low-risk patients to intermediate-risk patients beyond mPAP (*P* < 0.05) (Figure 5C).

## Discussion

The present study results reveal that in prevalent PAH patients, proximal PA has a significantly different remodeling according to the estimated risk derived from the ESC/ERS risk assessment table, leading to the RV-PA coupling impairment.

Risk stratification is dependent on several patient factors, including non-modifiable demographic factors (e.g., age, gender, and PAH etiology) and modifiable factors (functional capacity, RV function, and hemodynamic variables). We applied the ESC/ERS risk assessment tool employing seven modifiable variables largely influenced by RV adaptation to increased afterload (Galie et al., 2018). Each patient risk profile was obtained by grading each variable according to the risk categories and calculating a mean grade, rounded to the nearest integer. To our knowledge, this is the first study that links the estimated risk grade with PA structural and functional remodeling and RV-PA coupling.

**FIGURE 6:**
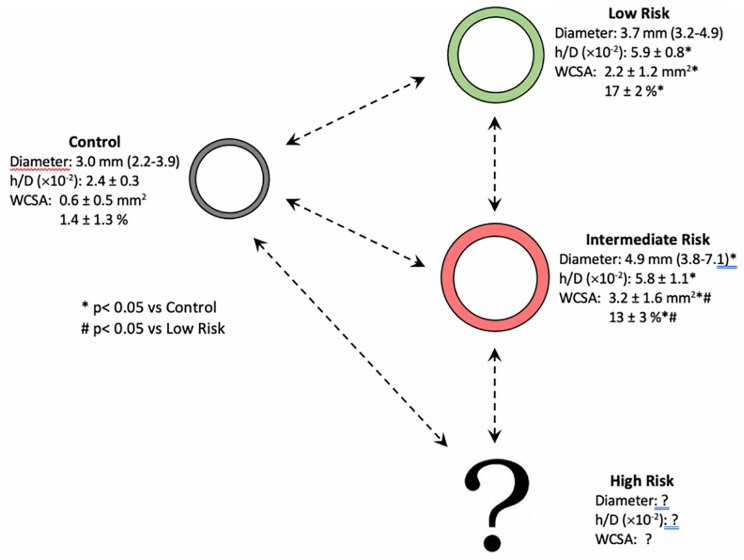
Proposed progression of the pulmonary arterial remodeling according to the stratification risk on prevalent PAH patients. Compared with controls, we may speculate that pulmonary arteries of PAH subjects could show an outward hypertrophic remodeling given by significant enlargement of vessel diameter, increased h/D ratio, and WCSA. The possibility of pathways with different trajectories is highlighted (h/D, wall thickness/lumen diameter ratio; PAH, pulmonary arterial hypertension; WCSA, wall cross-sectional area).

The arterial wall responds to prolonged transmural pressure and/or flow through geometrical, structural, and functional adaptation. The arterial remodeling tends to be vessel specific and takes place in order to restore stresses and strain to control levels. There are two major types of arterial structural remodeling depending on the change in vascular diameter and wall: inward or outward (decrease or increase in the diameter, respectively) and hypertrophic, eutrophic, and hypotrophic (increase, no change or decrease in the amount of wall components, respectively) (Mulvany, 1999). Compared with controls, we may speculate that PAs of PAH subjects could show an outward hypertrophic remodeling given by significant enlargement of vessel diameter, increased h/D ratio, and WCSA. Intermediate-risk patients had higher WCSA and lowered relative WCSA than low-risk subjects, maintaining the same h/D ratio, which allows a continuum in the remodeling process of the proximal PAs as the risk increases (Figure 6).

An interesting finding in this investigation is that the large PAs’ stiffening increases with the estimated risk. Stiffness is a mechanical behavior that describes the amount of force required to achieve a given deformation. The mechanical behavior of a vessel depends on the geometry and the wall viscoelastic properties. Since arteries are non-linear elastic materials, the increase in stiffness measured clinically may be due to the elevation in blood pressure, which increases vessel wall stretch and/or chronic remodeling-induced changes in extracellular matrix content (loss of elastin and increase in collagen content) and arterial geometry (Wang et al., 2017). We assessed local PA stiffness by different indices (Laurent et al., 2006). The intermediate-risk patients showed PA stiffness values similar to those published by Sanz et al. (2009). The arterial pulsatility, cross-sectional compliance coefficient, cross-sectional distensibility coefficient, and local PWV evaluated the elastic properties of the PA as a hollow structure and have more dependency on vessel geometry and intravascular pressure (extrinsic properties). The Einc and stiffness index β assess the inherent elastic properties of the arterial wall material and depend predominantly on the wall viscoelastic properties (intrinsic properties, mPAP independent) (Townsend et al., 2015; Chirinos et al., 2019). Although we cannot rule out an effect of stress/strain (extrinsic mechanism, mPAP dependent) on increased arterial stiffness in intermediate-risk compared to low-risk patients, wall remodeling-induced stiffening (intrinsic mechanism) plays a critical role. As it is widely known, the stiffness index β was proposed as the stiffness parameter, which does not depend upon blood pressures at the time of measurement due to the logarithmic transformation of the luminal distending pressure (Segers, 2017). Thus, the increase in stiffness index β observed in PAH patients, and the different values between low versus intermediate-risk subjects support an intrinsic change in PAs’ elastic properties. The significant upward shifted of the Einc values below and above the median values of the intermediate-risk group compared with low-risk patients would also be confirming the stiffer wall material of proximal elastic PAs beyond the indirect effect of raising the mPAP. Accordingly, there is growing evidence that suggests the association between the increase of vascular stiffness with an accumulation of collagen and loss of elastin in the proximal PAs during wall remodeling, making the vascular extracellular matrix an attractive potential therapeutic target not only for the treatment but also for the prevention of PAH (Thenappan et al., 2018). Lastly, the PAs Einc of our whole PAH cohort showed similar values of the normal systemic arteries at a pressure of 100 mmHg (∼500 kPa, 5 × 10^6^ dyn/cm^2^) (Westerhof et al., 2019).

By contrast to Bressollette et al. (2001) we found no differences in structural alterations between non-inferior and inferior pulmonary lobes in intermediate-risk patients. The use of a higher IVUS frequency probe could explain these anatomical differences. The worse functional remodeling in the segments of the non-inferior than inferior lobes with the same anatomical features could be explained by an increase in the collagen/elastin ratio of the arterial wall.

The significantly higher PA stiffness in the intermediate-risk group than low-risk PAH subjects was associated with a lower PAC. PAC depicts the pulsatile afterload in normal pulmonary circulation and contributes to approximately 25% of the total RV afterload (Saouti et al., 2010). The PAC values of our cohort showed similar values to the second (1.26–2.0 mL/mmHg; low-risk) and third quartiles (0.82–1.25 mL/mmHg, intermediate-risk) of the cohort studied by Mahapatra et al. (2006) who had 4-year mortality between 5 and 15%.

Although the proximal elastic PAs account for only 15–20% of the total PAC, they play a critical role in buffering pulsatile RV ejection and RV-PA coupling (Thenappan et al., 2016). The greater stiffness of PAs in the intermediate-risk group led to RV-PA uncoupling. All PA stiffness indices correlated with TAPSE/sPAP ratio. Therefore, the vascular stiffening of PA bed could be the main responsible for the RV-PA decoupling, either by an extrinsic mechanism (PA stress/strain stiffening, mPAP dependent) or intrinsic mechanism (wall remodeling-induced stiffening, mPAP independent). However, the possibility to differentiate between strain- and remodeling-induced PA stiffening in clinical settings by using different stiffness indices may lead to tailored treatments for PA stiffening in PH patients. Accordingly, whereas vasodilator therapy should reduce PA stiffness in PH patients with only strain-induced destiffening (extrinsic mechanism), inhibiting collagen accumulation or promoting collagen degradation would be more effective therapies in PH patients with both strain- and chronic wall remodeling-induced PA destiffening (extrinsic and intrinsic mechanism, respectively) (Golob et al., 2017).

The adaptation of RV function to loading is defined by a ratio of end-systolic to arterial elastances and has considerable reserve from normal values of 1.5–2 to 0.8, being able to detect the onset of RV failure (defined by excessive volume increase) in patients with PH (Tello et al., 2019a). By multivariate analysis, Tello et al. reported that TAPSE/sPAP is independently related to invasively measured Ees/Ea in severe PAH patients. Using receiver operating characteristic analyses and the Youden index, a TAPSE/sPAP cut-off value of 0.31 mm/mmHg identified patients with RV-arterial uncoupling defined by an Ees/Ea ratio < 0.805. Patients with TAPSE/sPAP < 0.31 mm/mmHg were at increased risk for clinical worsening and had an increase in mortality. They suggested the possible added value of TAPSE/sPAP in current risk assessment strategies for PAH (Tello et al., 2018). We could argue that low-risk patients with four subjects TAPSE/sPAP ratio below 0.3 mm/mmHg, had a RV-PA coupling reserve exhausted, illustrating the deleterious effect of arterial stiffening on the RV-PA coupling. Besides, TAPSE/sPAP of low-risk and intermediate-risk groups showed similar values to middle and low terciles of Tello et al. (2018) data, respectively.

The French low-risk approach analyzed the association between the number of low-risk criteria at baseline or achieved within 1 year of diagnosis and long-term prognosis using an abbreviated risk assessment tool. In our cohort, no patient had all variables in low-risk grade. The majority had five of seven variables in low-risk grade with arterial remodeling similar to patients with PH associated with interstitial lung disease candidates for lung transplantation (Domingo et al., 2017). Among the intermediate-risk subjects, six of eleven were in high-intermediate risk. Our findings strengthen the inappropriateness of the term “stable,” even for patients in the low-risk category who may have a significant PA remodeling with a RV-PA coupling reserve exhausted (Boucly et al., 2017; Hoeper et al., 2017; Kylhammar et al., 2018), and also supports not only low-risk profile as a pursue treatment goal but a novel candidate surrogate outcome in future clinical trials designs (Weatherald et al., 2018).

There are several limitations to this study. Although it is a small study cohort, the characterization of the PA remodeling of more than four segments per patient, obtaining the seven risk variables simultaneously, and the inclusion of prevalent and stable patients would overcome the low number of patients in interpreting the results. Several tools are currently available for assessing risk in PAH. Although the 2015 ESC/ERS PH risk table is based on expert opinion, do not include non-modifiable variables, do not use a weighting of variables, and do not work in all forms of PAH, a retrospective analysis of three major registries provided an independent validation of this approach and showed a clear difference in 5-year survival or transplantation-free survival, depending on risk stratification category at both baseline and first follow-up (Galie et al., 2018). We quantified the arterial stiffness in a pressure-independent way by the so-called stiffness index β. However, Spronck et al. (2017) have demonstrated that the common practice of using diastolic blood pressure and diameter values as a surrogate for the values at a reference pressure introduces pressure dependencies in the derived stiffness index β, questioning an intrinsic change in the arterial wall in patients with intermediate-risk. The upward shift of Einc values in function of the mPAP of intermediate-risk patients allows us to assume a real change in the viscoelastic properties of the arterial wall compared to low-risk subjects. Although the main PA stiffness indices can be assessed non-invasively by cardiac magnetic resonance, the advantage of IVUS is that it is performed concomitantly with RHC, and it provides data on vessel wall dimensions (WCSA and h/D).

## Conclusion

In prevalent PAH patients, the proximal PA stiffening impairs RV-PA coupling and is related to the simplified risk stratification. Despite a small sample size, our results show that proximal PA remodeling (intrinsic mechanism) is worse in intermediate-risk than in low-risk PAH patients, leading to RV-PA uncoupling, beyond the indirect effect of the mPAP (extrinsic mechanism). Probably, as the risk increase, the loss of elastin and the increase in the collagen content of the PA wall could explain the greater stiffness with less relative WCSA despite preserving h/D ratio. From a clinical perspective, it would be very interesting to explore the impact of therapy on the PA remodeling, RV-PA coupling, and risk grade of incident/naive PAH patients on their follow-up.

## Data Availability Statement

The raw data supporting the conclusions of this article will be made available by the authors, without undue reservation.

## Ethics Statement

The studies involving human participants were reviewed and approved by all procedures were approved by the Institutional Ethics Committee of the Hospital Universitari Vall d’Hebron (Barcelona). The patients/participants provided their written informed consent to participate in this study.

## Author Contributions

JG conceptualized the study objectives, performed the quantitative data analysis, interpreted data, and drafted the manuscript. ED participated in the study design, contributed to study coordination and data acquisition and preparation of the manuscript, and supervised the data collection. PT contributed to data analysis and preparation of the manuscript. CB and ML-M contributed to data analysis. SP-H contributed to statistical analysis of data. AR conceptualized the study objectives, obtained funding, and contributed to the study design, interpretation of data, and manuscript preparation. All authors contributed to the article and approved the submitted version.

## Conflict of Interest

The authors declare that this study received funding from GSK (GlaxoSmithKline), London, United Kingdom. The funder paid for the IVUS catheters used in the study. The funder was not involved in the study design, collection, analysis, interpretation of data, the writing of this article, or the decision to submit it for publication.

## Abbreviations

BNP: brain natriuretic peptide
dPAP: diastolic pulmonary artery pressure
Einc: incremental elastic modulus
Ep: strain-pressure elastic modulus
h/D: wall thickness/luminal diameter ratio
IVUS: intravascular ultrasound
LCSA: lumen cross-sectional area
mPAP: mean pulmonary artery pressure
6MWT: six-minute walking test
PA: pulmonary artery
PAC: pulmonary arterial capacitance
PAH: pulmonary arterial hypertension
PAOP: pulmonary arterial occlusion pressure
PH: pulmonary hypertension
pPAP: pulmonary artery pulse pressure
PVR: pulmonary vascular resistance
PWV: pulse wave velocity
RHC: right heart catheterization
RV: right ventricular
sPAP: systolic pulmonary artery pressure
TAPSE/sPAP: ratio of tricuspid annular plane systolic excursion to systolic pulmonary arterial pressure.
